# The Effect of Arctic Dust on the Retrieval of Satellite Derived Sea and Ice Surface Temperatures

**DOI:** 10.1038/s41598-018-28024-6

**Published:** 2018-06-27

**Authors:** R. F. Vincent

**Affiliations:** Royal Military College of Canada, Department of Physics and Space Science, Kingston, K7K 7B4 Canada

## Abstract

Large quantities of dust are transported annually to the Arctic, primarily from Asian deserts. The influx of dust into the polar environment changes the radiative properties of clouds while the deposition of dust onto ice and snow decreases the surface albedo. Atmospheric and surface dust may be identified with space borne radiometers by comparing infrared energy in the 11 μm and 12 μm regime. Between 2007 and 2017 satellite infrared data revealed persistent low-level dust clouds in the vicinity of Amundsen Gulf in the Western Canadian Arctic during the melting season. Evidence suggests that the subsequent deposition of atmospheric dust in the region affected the surface emissivity in the thermal infrared regime. As a result, satellite derived sea and ice surface temperature algorithms were rendered inaccurate in these areas. Moreover, the ubiquitous nature of dust in the region may play a role in the rapidly vanishing cryosphere.

## Introduction

The presence of aerosols in the Arctic environment is well established by researchers. A*rctic haze*, which was first identified in 1956^1^, is primarily the result of anthropogenic aerosol pollutants that originate from industrial areas^2,3^. Significant influxes of anthropogenic aerosols occur during the winter and spring as a result of enhanced transport mechanisms coupled with less efficient removal processes^4^. Anthropogenic aerosols affect the microphysical structure of arctic clouds, leading to enhanced surface longwave fluxes that are comparable to established greenhouse gases^5,6^. Non-anthropogenic aerosols, such as mineral dust, are also introduced into the Arctic atmosphere. Locally, retreating ice masses expose fine sediments that can lead to dust storms, particularly in the autumn^7^. However, the major source of naturally occurring aerosols in the polar region is through the transport of dust from southern latitudes. Globally, the entrainment of desert particulates is the most significant source of atmospheric mineral dust, contributing to 75% of all aerosols^8^. The majority of dust transported to the Arctic originates in Asian deserts with smaller contributions from the Sahara Desert^9,10^. The peak in arctic dust concentration occurs during the spring, which coincides with dust storms in the Gobi and Takliman Deserts^9,11^. It is estimated that dust is transported directly to the Arctic in 25% of Asian dust storm events^12^. Accelerated dryland expansion as a result of climate change provides additional sources of dust^13^. Overall, approximately 6.5 million metric tons of dust are deposited every year between 60°N and 90°N^14^.

In recent decades it has been observed that near surface air temperatures are increasing more rapidly in the Arctic than the global average, a process known as Arctic amplification^15,16^. Although the underlying mechanisms are not fully known, changes in atmospheric and oceanic circulation^17,18^, influences of cloud cover and water vapour^19,20^, as well as the reduction in surface albedo^21–23^ figure prominently in Arctic warming discussions. With respect to the influx of dust to the Arctic, these light absorbing aerosols are deposited on snow and ice, reducing the surface albedo and leading to accelerated melt rates^24,25^. Dust particles also change the radiative properties of clouds by inducing ice nucleation and cloud condensation nuclei^9,26^. Calculations of radiative forcing based on reconstructions of dust concentrations for the Holocene and Last Glacial Maximum suggest that the impact of dust aerosols in the Arctic is underestimated in Arctic amplification models^27^.

**Figure 1:**
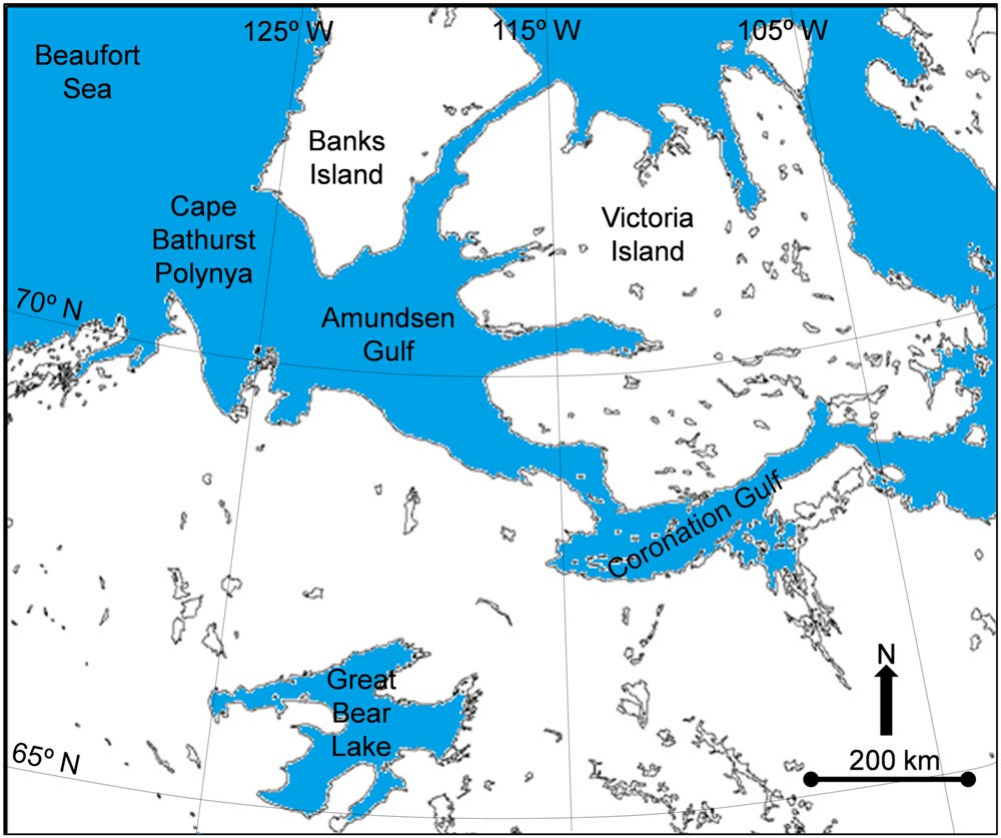
The research focuses on satellite images of Amundsen Gulf and surrounding waters, including Cape Bathurst Polynya and Great Bear Lake. (Map created with Harris Geospatial ENVI 5.3 software, http://www.harrisgeospatial.com).

This research focuses on the observed persistence of dust clouds in the Canadian Western Arctic and the subsequent apparent deposition of dust on seawater and ice surfaces. In the case of deposition, the particulates modify the surface emissivity in the thermal infrared (IR) regime. Application of the Composite Arctic Sea Surface Temperature Algorithm (CASSTA)^28^ to Advanced Very High Resolution Radiometer (AVHRR) imagery in the vicinity of Amundsen Gulf (Fig. 1) during the melting season showed significant anomalies that were initially assessed as artifacts. Further investigation of sea and ice temperatures in the region revealed similar anomalies in the satellite record, leading to the conclusion that the observation was a physical manifestation of atmospheric and surface properties.

## Methods

### Satellite Detection of Dust

**Figure 2:**
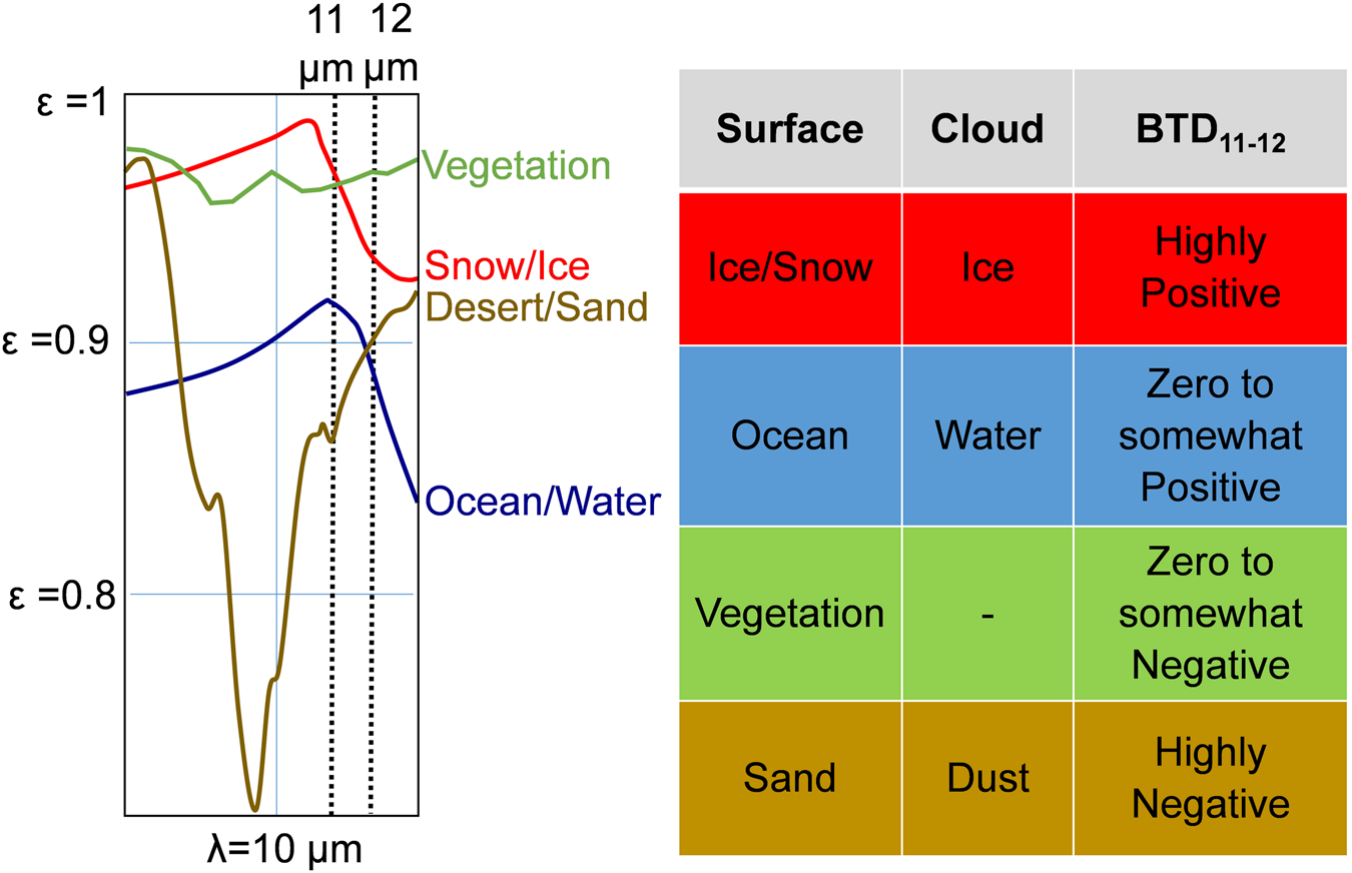
Angularly averaged emissivity (*ε*) of snow/ice, ocean/water, desert/sand and vegetation for thermal IR wavelengths^29^ and BTD_11–12_ for surface and cloud types^31^. The resulting difference in brightness temperature between 11 μm and 12 μm channels, or BTD_11–12_, allows the differentiation between surface and cloud types. The emissivity of bare land is a function of rock or soil type, though BTD_11–12_ is commonly zero to highly negative^29,31^. In this study, land areas consisting of tundra, generally showed highly positive BTD_11–12_. Optically thin dust cloud BTD_11–12_ may be affected by the underlying terrain while surface signatures are influenced by clear sky absorption of thermal IR energy by atmospheric water vapor or ice crystals.

Naturally occurring materials such as ice, snow, water and sand are selective emitters in which the emissivity is a function of wavelength^29,30^. The difference in emissivity between wavelengths can help to identify a type of cloud or surface viewed by a satellite radiometer. Space based sensors that operate in the thermal IR regime utilize bands centered on 11 μm and 12 μm, both of which correspond to atmospheric windows and the region of peak radiation emission of the Earth. Figure 2 illustrates the change in emissivity between 11 μm and 12 μm for vegetation, snow/ice, desert/sand and ocean/water surfaces. For snow/ice there is a sharp drop in emissivity while the opposite is true for desert surfaces. There is a slight decrease for water surfaces and slight increase for vegetation. Generally, a satellite radiometer will record different brightness temperatures for the two channels as a result of the different emissivity values for these wavelengths. The Brightness Temperature Difference between the 11 μm and 12 μm channels (BTD_11–12_) is highly positive for ice, somewhat positive for water, somewhat negative for vegetation and highly negative for sand^31^. The BTD_11–12_ for ice, water and desert surfaces can be extended to an ice cloud, water cloud and dust cloud respectively. Hence, the differentiation between surface and cloud types is possible through the analysis of satellite BTD_11–12_ data.

Dust clouds may be identified from space using thermal IR channels on the Moderate Resolution Imaging Spectroradiometer (MODIS) on NASA’s Terra satellite. The sensor has 36 channels, including Channel 31 centered at 11 μm (10.8 μm to 11.3 μm) and Channel 32 centered at 12 μm (11.8 μm to 12.3 μm). Spatial resolution for the thermal IR channels is 1 km. Using MODIS BTD_11–12_ values, the extent and intensity of a dust event can be ascertained. Dust events are identified when BTD_11–12_ < −0.5 K in cloudy regions^31^. In the development of the algorithm, it was determined that the surface contributed −0.5 K to the BTD_11–12_, necessitating a threshold at that value. The intensity of the dust event may be determined by taking the difference of MODIS Channel 29 centered at 8.5 μm (8.4 μm to 8.7 μm) and Channel 31, or BTD_8–11_. In a region that meets the parameters of a dust event, BTD_8–11_ > 0 represents a relatively strong dust region while BTD_8–11_ < 0 signifies a relatively weak dust region^31^. A comparison of concurrent (±1 hour) AVHRR and MODIS imagery in this study demonstrated that AVHRR Channel 4 (10.3 μm to11.3 μm) minus Channel 5 (11.5 μm to 12.5 μm), with a spatial resolution of 1.1 km, is comparable to MODIS in identifying BTD_11–12_ dust signatures.

In the arctic environment over areas of ice, snow, water and tundra where BTD_11–12_ > 0, there is no requirement for a threshold of −0.5 K. In this study, a cloud with a negative BTD_11–12_ is classified as a dust cloud, while any ice, snow or water surface exhibiting a negative BTD_11–12_ under clear skies implies that there is layer of deposited material on top. Space based radiometers measure the skin temperature of the sea surface, which is less than 0.1 mm, so a very thin layer can return a signature that is indicative of a desert instead of a body of water or ice sheet. While it is possible that a substance other than mineral dust is causing the negative BTD_11–12_ signature, such as anthropogenic aerosols, there is nothing in the literature to support this theory. *In situ* sampling will solve the issue of chemical composition and provenance of the material. Regardless, it is a known certainty that a negative BTD_11–12_ cannot be produced by a pure water, ice or snow surface.

### Sea and Ice Surface Temperature Retrieval

Clear skies are required for satellite-derived sea and ice surface temperatures. Satellite sea surface temperature (SST) algorithms for temperate oceans utilize BTD_11–12_ to determine clear sky atmospheric absorption between the sensor and sea surface. The presence of water vapor causes a positive BTD_11–12_ so a more positive value leads to a greater correction to the main estimator at 11 μm (BT_11_) to account for this absorption. AVHRR SST algorithms generally take the form,

1 $${\rm{S}}{\rm{S}}{\rm{T}}=a+b{{\rm{B}}{\rm{T}}}_{11}+c({{\rm{B}}{\rm{T}}{\rm{D}}}_{11{\textstyle \mathrm{ \mbox{-} }}12})+d[{{\rm{B}}{\rm{T}}{\rm{D}}}_{11{\textstyle \mathrm{ \mbox{-} }}12}(\sec \theta -1)],$$

where *a*, *b*, *c* and *d* are coefficients normally based on a regression analysis of concurrent satellite and *in situ* data, and *θ* is the sensor zenith angle. The final term in the algorithm accounts for amplified atmospheric absorption resulting from increased path length to the satellite sensor. For the computation of SSTs a moist atmosphere has a BTD_11–12_ ≥ 0.7 K while a dry atmosphere corresponds to BTD_11–12_ < 0.7 K^32^. Since a positive BTD_11–12_ is expected, negative values will return an inaccurately cool SST. An ice surface temperature (IST) algorithm takes the same form as equation (1), but in this case the BTD_11–12_ term accounts for a number of factors including modeled directional snow emissivities^33^. An IST BTD_11–12_ term should be positive, with a negative value returning incorrect cooler temperatures. In the case of dust, which has a lower emissivity than water and ice for BT_11_ (Fig. 2), the error of the retrieved temperature using equation (1) is further amplified.

### Surface Temperature Retrieval using CASSTA

The Arctic is a dry climate, with an annual mean distribution of specific humidity near the surface of 1 g kg^−1^, compared to 18 g kg^−1^ in equatorial regions^34^. In accordance with the paradigm for temperate water SST algorithms, atmospheric BTD_11–12_ over Arctic waters should be low; however, high values are observed, particularly during the colder months^35^. Inflated atmospheric BTD_11–12_ in the Arctic is a function of clear sky ice crystals that absorb more 12 μm (BT_12_) energy than water vapor^35^. Arctic BTD_11–12_ values are highest in the winter, with values exceeding 2 K in regions of ice fog in the vicinity of leads, and reducing to approximately 0.5 K during the summer^35^. As a result, temperate water algorithms overestimate Arctic SSTs by 2 to 3 K^28^. CASSTA, which was developed with AVHRR and concurrent *in situ* data, combines three different algorithms to determine the temperature of seawater, marginal ice zones and ice, each of which is designated by the BT_11_ value. A single channel (BT_11_) is used to determine Arctic SST to avoid high BTD_11–12_ brought about by enhanced BT_12_ absorption by atmospheric ice crystals, which is not statistically related to absorption of the main estimator. Marginal ice zones use a weighted average between a standard IST and the Arctic SST, while ice regions use the IST algorithm^28^. In the case of dust deposition on the surface, the Arctic SST will return an inaccurately cool temperature as a result of the lower emissivity of dust compared to water for BT_11_ (Fig. 2).

### Satellite Data for the Study

The Arctic is a challenging environment to retrieve satellite derived SSTs. With the exception of polynyas, open water is limited to the melting season, which is characterized by the persistence of arctic stratus^36^ with estimates of July cloud cover exceeding 90%^37^. In this study METOP-A and National Oceanic and Atmosphere Administration (NOAA) AVHRR satellite imagery was initially used to determine BTD_11–12_ in the Amundsen Gulf region. AVHRR was chosen for the initial search since CASSTA could be applied to these images to evaluate the magnitude of surface temperature discrepancies. Anomalies were identified as a negative BTD_11–12_ in areas of water and ice that appeared cloud free. The initial search for anomalies focussed on June to August for the years 2007 to 2017. One to three images were available per day between 17Z and 21Z of which about 20% were sufficiently cloud-free for further examination. Negative BTD_11–12_ anomalies were detected at least once for June, July and August for every year from 2007 to 2017. Most imagery showed negative BTD_11–12_ in optically thin, low lying clouds in the vicinity of Amundsen Gulf. Once anomalous areas were identified with AVHRR, a search for MODIS images for these days was carried out. Appropriate MODIS images were thermally calibrated, georeferenced and the dust cloud detection algorithm applied. Random AVHRR and MODIS images selected for the melting season between 2002 and 2006, revealed similar results to the 2007 to 2017 timeframe. While the study concentrated on the melting season, a sampling of AVHRR and MODIS imagery during winter and early spring in 2016 showed extensive areas of negative BTD_11–12_. For example, AVHRR imagery of the study area had atmospheric or surface negative BTD_11–12_ every day in March 2016.

### Data availability

All data is available on-line and free of charge. AVHRR data is available at the National Oceanic and Atmospheric Administration (NOAA) Comprehensive Large Array-data Stewardship System (CLASS). MODIS data can be found at National Aeronautics and Space Administration (NASA) Level-1 and Atmosphere Archive and Distribution System (LAADS).

## Results

### AVHRR Imagery

CASSTA was developed in the North Water Polynya (NOW) situated between Elsmere Island and Greenland (78 N 76 W). Initially, the purpose of this study was to compare CASSTA to temperate ocean SST algorithms in more southern regions of the Arctic. The application of these algorithms to the Amundsen Gulf region (70 N 120 W) focussed on a relatively cloud free period 1 to 12 July 2016. During this timeframe, there were instances when temperate ocean SST algorithms returned cooler surface temperatures than CASSTA, which is not possible under normal atmospheric conditions. Further investigation showed that these anomalies were the result of negative BTD_11–12_. Examination of 210 AVHRR satellite images for June to August 2016 revealed the following observations.

- A persistence of low-level, optically thin clouds with BTD_11–12_ ranging from 0 to −1 K.
- A regular occurrence of optically thick clouds with BTD_11–12_ ranging from 0 to +2 K.
- Land surfaces, consisting primarily of tundra, showed a strong positive BTD_11–12_.
- Under apparent clear skies, ice and seawater in the Amundsen Gulf region and Great Bear Lake periodically showed negative BTD_11–12_ that is not characteristic of these surface types.

**Figure 3:**
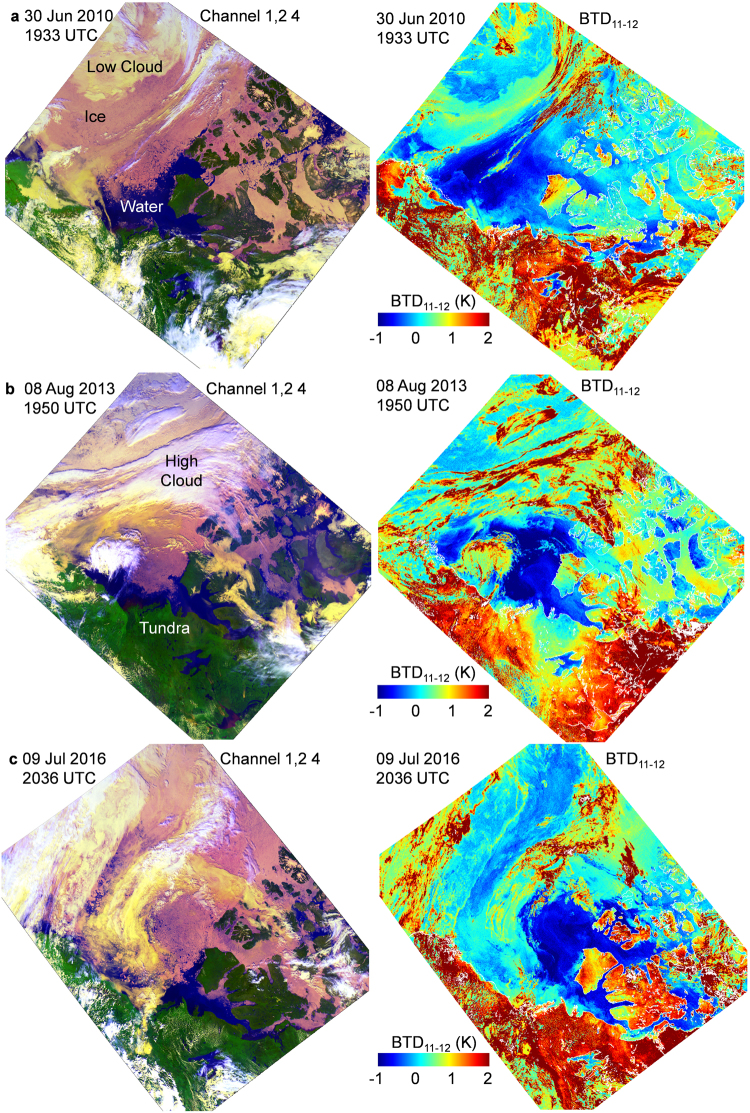
AVHRR Channels 1 (0.58 μm to 0.68 μm), 2 (0.725 μm to 1.00 μm), 4 and corresponding BTD_11–12_ mappings are shown for (**a)** 30 Jun 2010 (**b**) 08 Aug 2013 and (**c**) 09 Jul 2016. Areas of highly negative BTD_11–12_ over ice and water are approximately 400,000 km^2^. The BTD_11–12_ signature in these areas is not representative of water or ice surfaces and is in stark contrast to the expected positive BTD_11–12_. (Images created with Harris Geospatial ENVI 5.3 software, http://www.harrisgeospatial.com).

**Figure 4:**
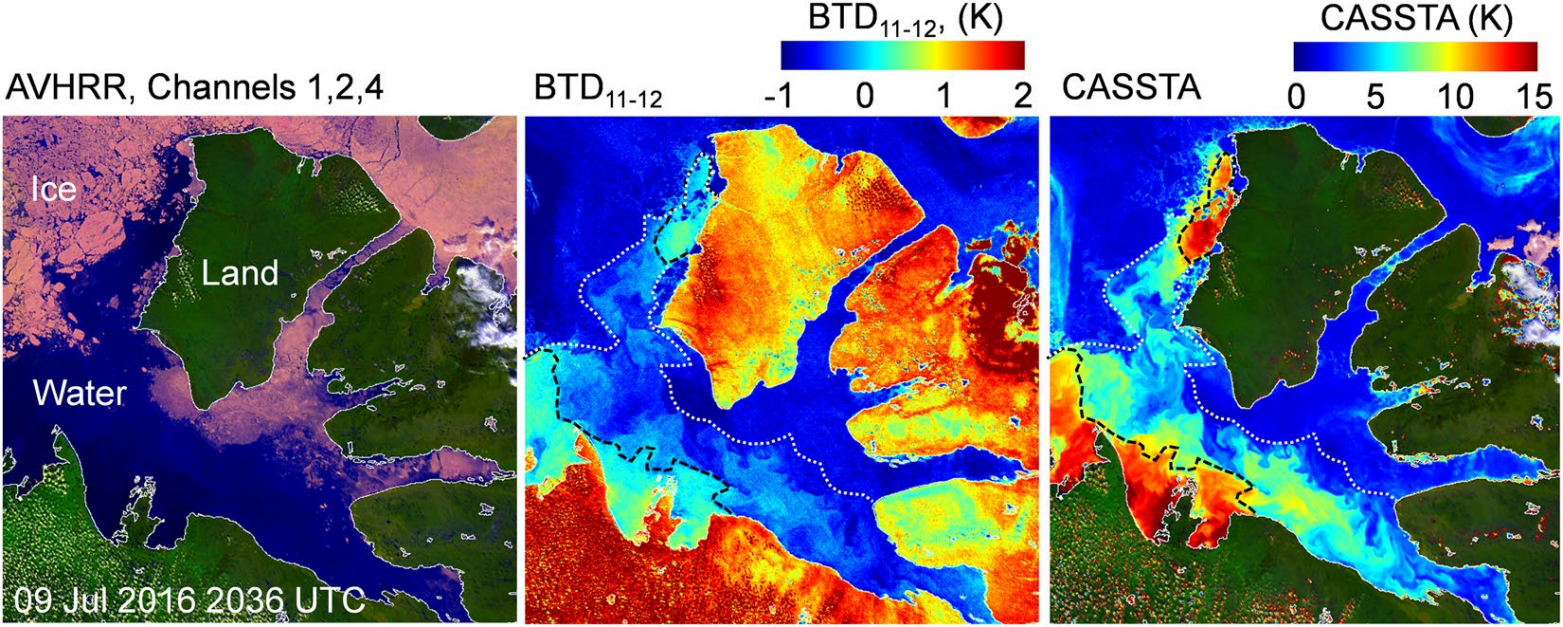
AVHRR Channels 1, 2, 4 and corresponding BTD_11–12_ and CASSTA mappings are shown for 09 July 2016. The white dotted lines indicate approximate ice extent while the black dashed lines represent the boundary between negative BTD_11–12_ and positive BTD_11–12_. SST values for negative BTD_11–12_ are significantly lower, indicating that there is a reduction in emissivity in these regions. (Images created with Harris Geospatial ENVI 5.3 software, http://www.harrisgeospatial.com).

The initial CASSTA study of the area was expanded to include other years. Over 2,300 AVHRR for the Amundsen Gulf region were examined for June to August 2007 to 2017. Similar anomalies were detected every year to varying degrees, the detection of which was often hindered by clouds. Figure 3 shows the extent of negative BTD_11–12_ for selected AVHRR scenes in 2010, 2013 and 2016. A closer inspection of Amundsen Sound for the 09 July 2016 image shows a mixture of positive and negative BTD_11–12_. Figure 4 compares the BTD_11–12_ mapping and CASSTA retrieval for this day. Areas of open water with a positive BTD_11–12_ are 6 K to 10 K warmer than water with negative BTD_11–12._ Since CASSTA uses BT_11_ solely to calculate SST, this result implies that negative BTD_11–12_ regions have reduced emissivity in the 11 μm regime. The negative BTD_11–12_ in concert with lower temperatures, is consistent with the properties of dust.

### MODIS Imagery

**Figure 5:**
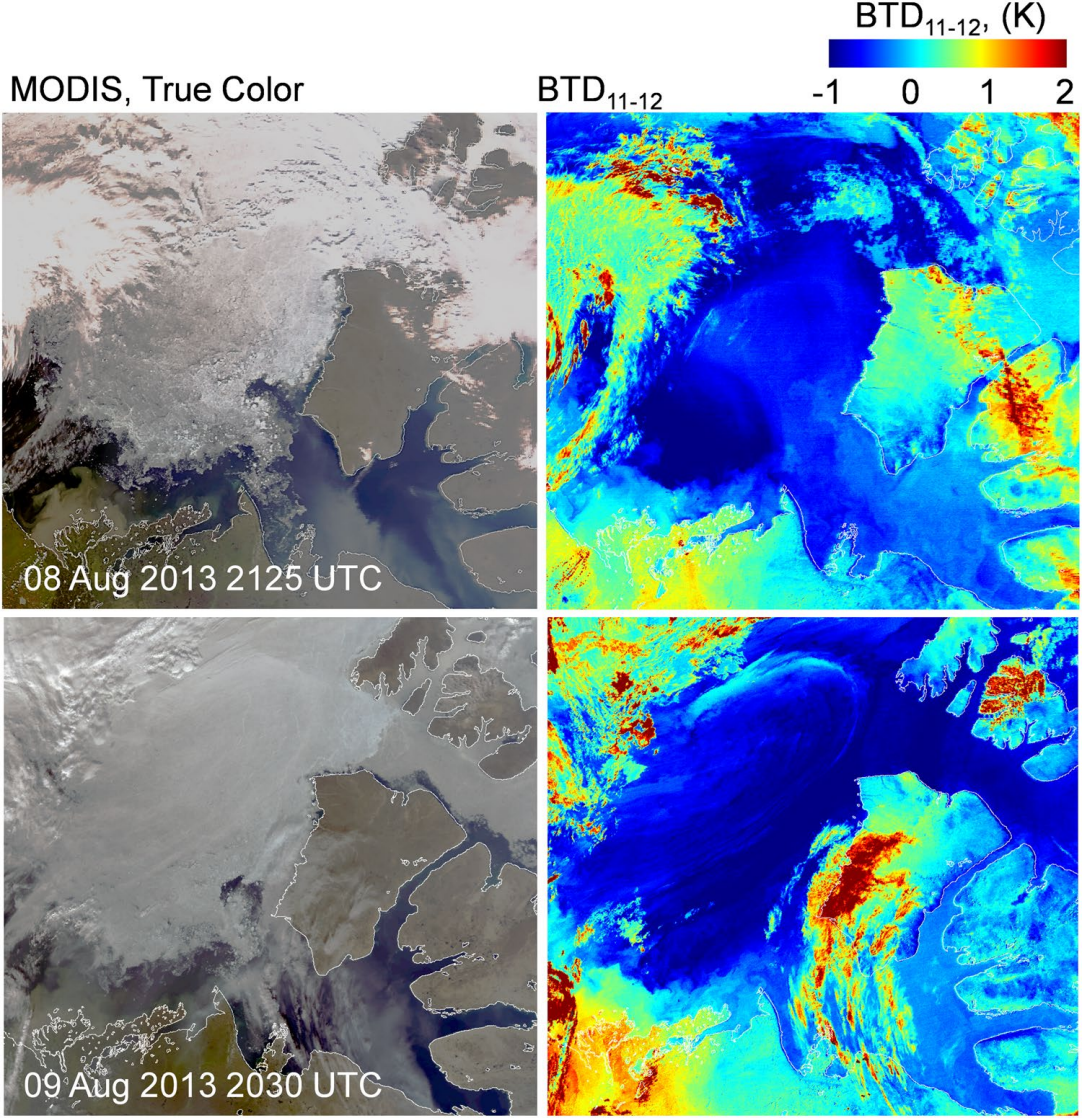
MODIS true color images on the left are shown for 08 and 09 August 2013 with corresponding BTD_11–12_ mappings on the right. A dust cloud is identifiable in dark blue for both days. Portions of these clouds meet the criteria for a strong dust event (BTD_11–12_ < 0, BTD_8–11_ > 0). Higher clouds, identified by positive BTD_11–12_, block out portions of the lower dust cloud for both images. The surface is visible through much of the dust cloud. (Images created with Harris Geospatial ENVI 5.3 software, http://www.harrisgeospatial.com).

The dust detection algorithm was applied to MODIS imagery to corroborate the AVHRR analysis. In all cases of concurrent AVHRR/MODIS imagery there was agreement between the two sensors. The thermal channels for MODIS are 0.5 μm wide compared to 1 μm for AVHRR, which generally resulted in more negative BTD_11–12_ dust signatures for that sensor. Dust clouds were generally observed as optically thin, low lying features as illustrated in Fig. 5. The addition of the 8 μm channel on MODIS allowed the intensity of the dust event to be evaluated using BTD_8–11_. The majority of dust clouds tested were classified as relatively weak, although there were examples of strong dust events that persisted for extended periods.

**Figure 6:**
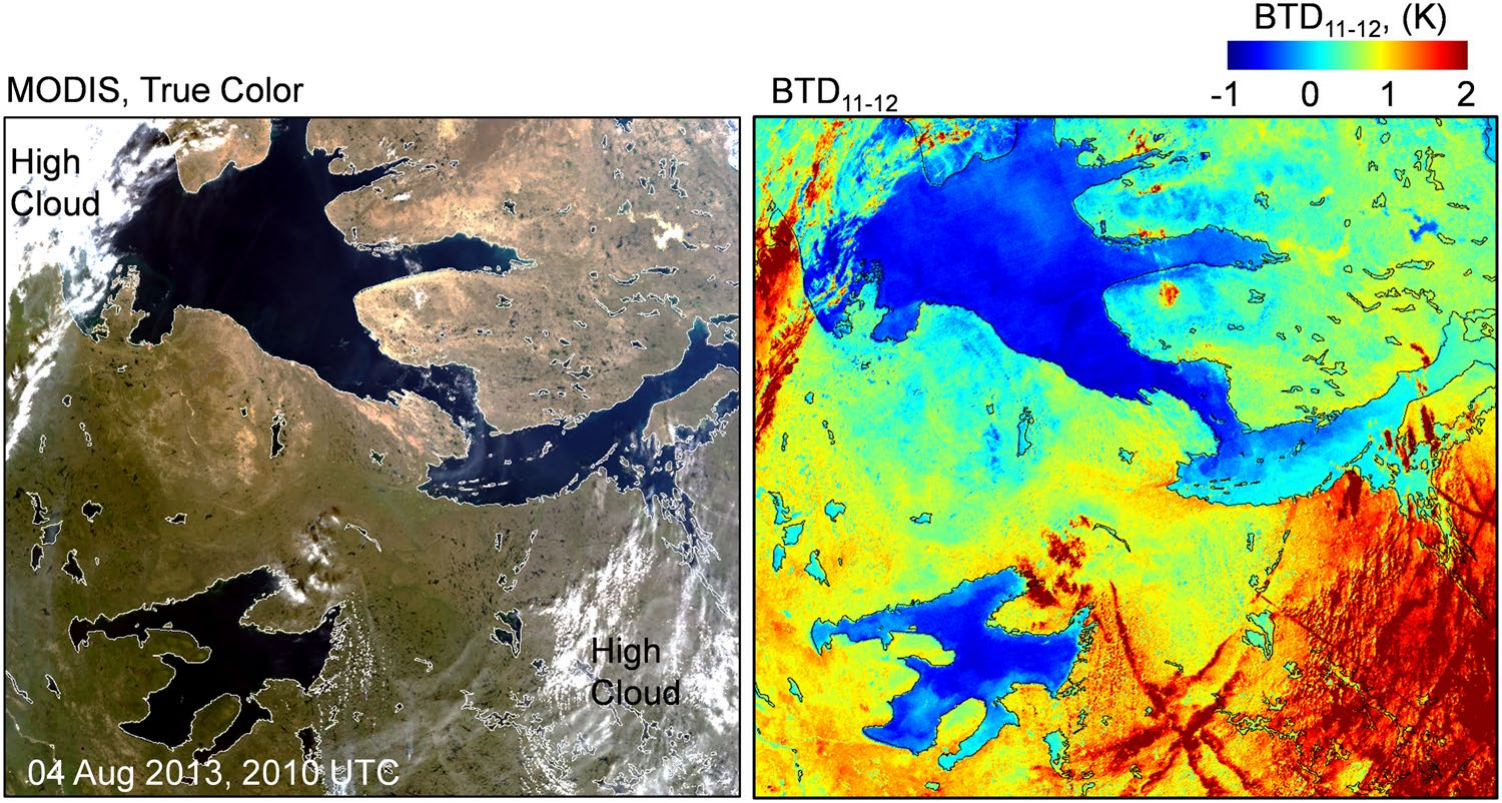
A MODIS true color image and the corresponding BTD_11–12_ mapping is shown for 04 Aug 2013. The negative BTD_11–12_ of the water surfaces indicate that the signature cannot be the result of water, suggesting that a material such as dust is coating the surface. Textural difference in the land between Great Bear Lake and Amundsen Gulf in the true color image correspond to relatively low BTD_11–12_. This may be the result of dust deposition or the natural state of the surface for August. Highly positive BTD_11–12_ indicate high ice clouds. (Images created with Harris Geospatial ENVI 5.3 software, http://www.harrisgeospatial.com).

Similar to AVHRR, the MODIS imagery showed ice and water surfaces with negative BTD_11–12_ under apparent clear skies. It is possible that optically thin dust clouds close to the surface are creating a signature that mimics one of dust deposition. For these clear sky cases, the negative BTD_11–12_ follows the coastline, while the adjacent land surfaces generally show highly positive BTD_11–12_. On many occasions during the study period Great Bear Lake, approximately 200 km south of Amundsen Gulf, also displayed negative BTD_11–12_ under apparent clear skies. In these instances, the land between the two bodies of water showed positive BTD_11–12_ values with no visual cues of a dust cloud. Figure 6 compares a MODIS image of Great Bear Lake and Amundsen Gulf to the BTD_11–12_ mapping of the same scene. The evidence suggests that dust deposition has occurred on Great Bear Lake and Amundsen Gulf. Some of the land close to Amundsen Gulf is less positive, which may be the result of dust deposition or the natural state of the surface for August.

**Figure 7:**
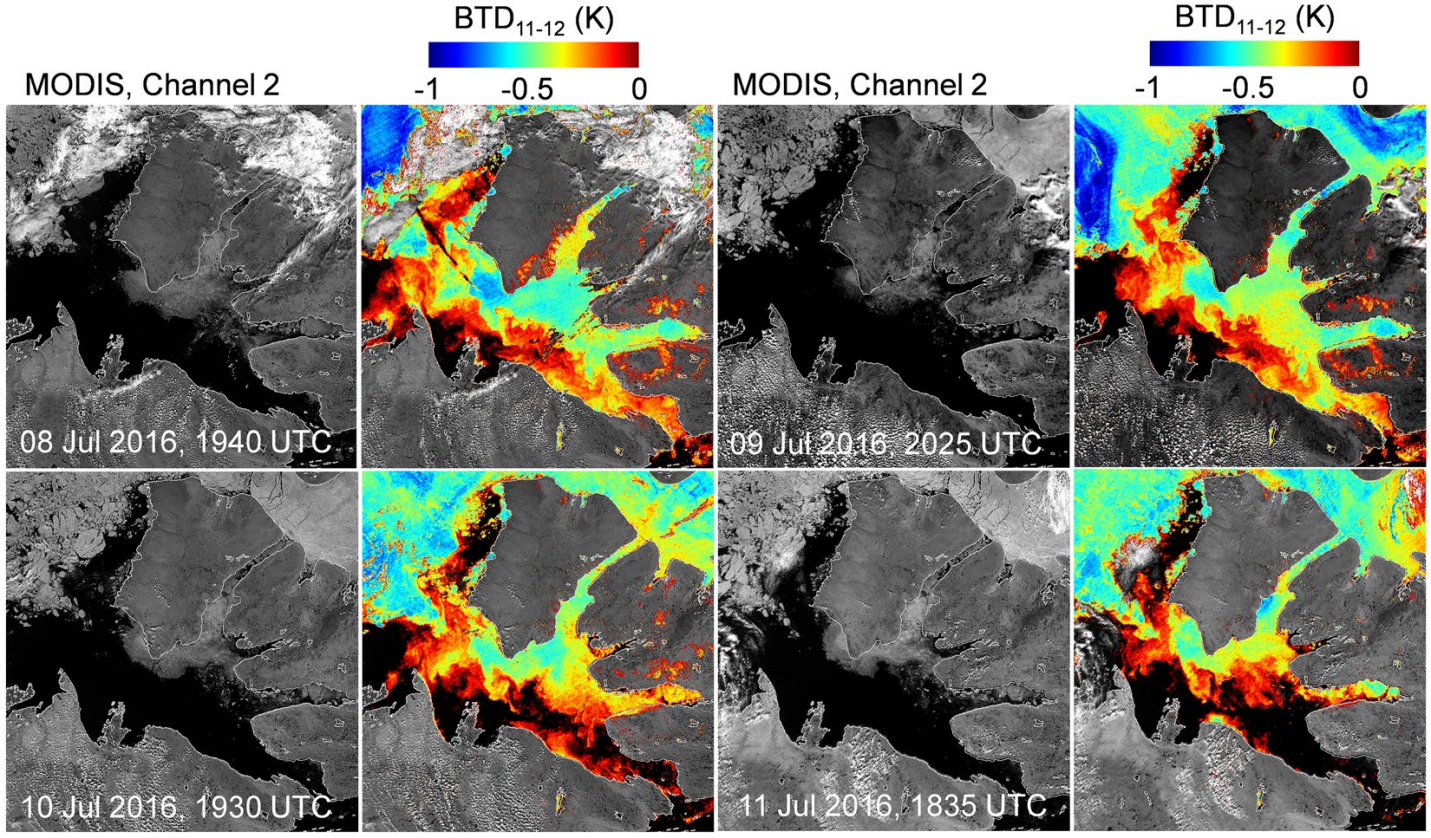
MODIS images of Amundsen Gulf region for 08 to 11 July 2016. A Channel 2 (0.841 μm to 0.876 μm) with a corresponding negative BTD_11–12_ overlay to the right is shown for each day. A dust signature persists on the water (to the south) and ice (to the north) for at least four days. Clouds prevented observation of the onset and dissolution of the phenomenon. The more negative BTD_11–12_ signatures appear on the ice, which supports the hypothesis that it is a surface phenomenon. (Images created with Harris Geospatial ENVI 5.3 software, http://www.harrisgeospatial.com).

The formation, evolution and dissolution of anomalous sea and ice surface BTD_11–12_ is difficult to observe with satellite data as a result of persistent cloud cover. While there are cases of the phenomenon dispersing within 24 hours, there are also examples in the satellite record that demonstrate the potential for anomalies to last for extended periods. Figure 7 shows four consecutive days in July 2016 of negative BTD_11–12_ on the surface of Amundsen Gulf. Clouds obstructed clear observation of the onset and dissolution of the phenomenon, which could be seen intermittently through breaks in the cloud for twelve days. In this series of images, the BTD_11–12_ of ice is more negative than that of water. If the area was covered by an optically invisible dust cloud, the opposite would be true since the positive contribution of BTD_11–12_ from an ice surface is greater than that of water. An explanation for this observation is that dust can collect on ice in greater density than a water surface that moves under the influence of wind and current.

### Winter Imagery

**Figure 8:**
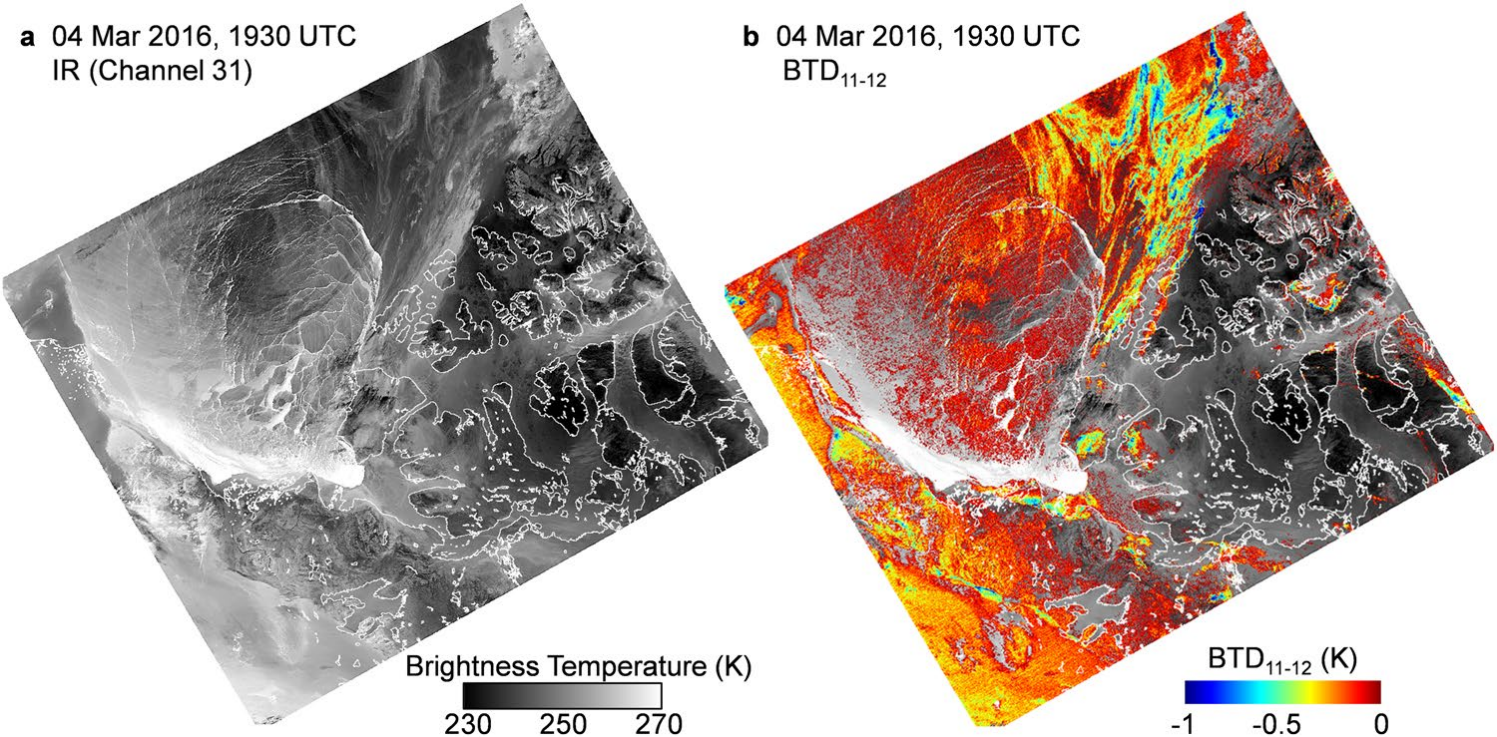
(**a**) MODIS IR image (Channel 31) of Amundsen Gulf region is shown for 04 March 2016. Bright areas indicate warmer temperatures. The warmest areas are leads to the northwest in the Beaufort Sea and open water in Cape Bathurst Polynya. The ice surface over the Beaufort Sea is visible through optically thin clouds. (**b**) A mapping of negative BTD_11–12_ overlaid on the same scene reveals an extensive dust signature, both in the clouds and potentially on the ice. The bright areas in the vicinity of Cape Bathurst Polynya have BTD_11–12_ values as high as 2 K. This may be the result of ice fog dominating the BTD_11–12_ signature in areas of open water^35^. (Images created with Harris Geospatial ENVI 5.3 software, http://www.harrisgeospatial.com).

Dust clouds identified by negative BTD_11–12_ on MODIS images are readily apparent during the winter months in the Arctic since the underlying icescape is highly positive. Figure 8a is a MODIS thermal image (Channel 31) of a characteristic winter scene of Amundsen Gulf region with brighter areas indicating warmer brightness temperatures. There is a small amount of open water in the vicinity of Cape Bathurst Polynya that gives the warmest temperature, while leads in the ice pack also show elevated thermal signatures. Figure 8b shows the corresponding BTD_11–12_ mapping with negative values superimposed on the thermal image. Similar to the melting season, the dust clouds identified by negative BTD_11–12_ are low-level features that are blocked by higher ice clouds that exhibit highly positive BTD_11–12_. Clouds at any level may potentially contain dust aerosols that have been coated with ice and subsequently give the highly positive BTD_11–12_ signature of an ice cloud. In many cases the regions identified as dust clouds are optically thin, appearing as a haze that allows the ice features to be viewed below (Fig. 3a). The signature of dust clouds in the winter are generally less negative than those observed in the warmer months as a result of the contribution of the highly positive surface BTD_11–12_. In the case of open water, a highly positive ice fog signature^35^ may overwhelm the negative BTD_11–12_ dust signature (Fig. 3b). In apparent cloud free areas, some ice exhibits negative BTD_11–12_, suggesting dust deposition on the surface (Fig. 3b). Although this study did not focus on the colder months, dust clouds were commonly observed in winter satellite imagery of the region.

## Discussion

In this study significant dust clouds, identified by negative BTD_11–12_ using satellite radiometers, were detected in the in the Amundsen Gulf region during the melting season. Dust signatures were also observed on ice and water surfaces under apparent clear skies, suggesting dust deposition. Surrounding land surfaces in the region generally did not show negative BTD_11–12_, although values may have been less positive as a result of dust deposition. The presence of dust prevents accurate SST and IST retrieval. Regardless of whether the phenomenon is a surface or a near-surface feature, the problem for satellite surface temperature retrieval using AVHRR remains the same.

Previous studies of Arctic SSTs in the NOW^28,38^, were not hampered by dust clouds or dust deposition on the surface. The observed persistence of dust in the vicinity of Amundsen Gulf is notable since the apparent deposition of particulates changes the emissive properties of the surface. In this study, the application of CASSTA to negative BTD_11–12_ regions led to an underestimation of 5 K to 10 K as a result of the lower emissivity of dust compared to water. The application of temperate water SSTs applied to the same area results in an even greater error as a result of the BTD_11–12_ term in the algorithm that is designed to account for absorption by atmospheric water vapor. In the case of a negative BTD_11–12_ this corrective term, which is supposed to add to the main estimator (BT_11_), will erroneously reduce the value by an additional 1 K to 5 K depending on the BTD_11–12_ value and sensor zenith angle. These are significant errors for SST algorithms, which are projected to determine sea surface skin temperatures with an accuracy of better than 0.5 °C^39^. A mask could be used to identify and avoid areas of negative BTD_11–12_; however, in some cases dust may simply reduce highly positive BTD_11–12_ generally observed in the Arctic to values that are still above zero but erroneous. As such, significant errors could still be propagated in the data.

An IST algorithm was not utilized in this study, since summer ice in Arctic waters typically exceed the BT_11_ threshold (271 K) in which the SST portion of the CASSTA is employed. This aspect of the algorithm leads to better ice differentiation during the summer months^28^. During colder months an IST algorithm will potentially return even cooler anomalies than water as a result of having a higher emissivity differential compared to dust (Fig. 2). Additionally, the BTD_11–12_ component of the algorithm will further increase the error. The observed dust in winter scenes may also promote the formation of ice fog in the vicinity of leads and polynyas as a result of ice nucleation. Ice fog is difficult to detect with satellite imagery and prevents the determination of surface temperature^28^. Application of an IST algorithm to these regions will overestimate the surface temperature as a result of inflated BTD_11–12_ (>2 K) that is related to atmospheric ice crystals^35^. It should be noted that the ice fog BTD_11–12_ signature is a function of enhanced IR absorption in the 12 μm regime.

While atmospheric and surface dust is problematic for SST and IST satellite retrieval, the ubiquitous nature of the phenomenon highlights issues that extend beyond the discipline. It is estimated that the introduction of mineral dust into the Arctic climate plays a role in Arctic Amplification^9^ and the subsequent loss of ice. On the other side of the globe, surface air temperatures in Antarctica have not risen to the same extent as the Arctic and ice extent has trended upward since 1979^40^. Approximately 16 times more dust is transported to Arctic latitudes compared to equivalent Antarctic latitudes^14^ as a result of fewer dust sources in the southern hemisphere coupled with unfavourable wind trajectories^41^. This suggests that the reduction in dust aerosols transported to Antarctica may be a factor in the disparity between climatic trends of the two Polar Regions. In the Arctic, the greatest decline in sea ice over the past two decades is the Western Arctic, which is losing ice at a faster rate than predicted by models^42^. The high frequency of dust events observed in the vicinity of Amundsen Gulf may be a contributing factor to the rapid disappearance of the cryosphere in the region.
